# The Detection of Early Reading Performance and Its Relationship with Biopsychosocial Risk Factors in the Study of Learning Difficulties

**DOI:** 10.3390/ejihpe12080084

**Published:** 2022-08-20

**Authors:** Cristina Quiroga Bernardos, Santiago López Gómez, Patricia María Iglesias Souto, Rosa María Rivas Torres, Eva María Taboada Ares

**Affiliations:** Department of Developmental and Educational Psychology, Faculty of Psychology, University of Santiago de Compostela, 15782 Santiago, Spain

**Keywords:** reading predictors, reading difficulties, developmental dyslexia, learning disabilities, alphabet knowledge, phonemic awareness, maternal risk, perigestational, biopsychosocial factors, psycholinguistic abilities

## Abstract

The study of the multiple processes involved in learning how to read can contribute towards the early detection of good and bad readers. However, it is necessary to take into consideration different biopsychosocial risk factors (pre- and perigestational, neonatal, medical, developmental and family-related) that may have a significant impact on neurodevelopment, producing atypical cognitive development that could lead to the presence of reading difficulties. The objective of this study was to identify the main psycholinguistic abilities involved in the early reading performance and analyse their relationship to biopsychosocial risk factors. A total of 110 subjects between the ages of 4 and 7 years old and enrolled in state-run schools in Spain participated in the study. Significant correlations were found between different psycholinguistic abilities and certain biopsychosocial risk factors (having had hyperbilirubinemia, having obtained a score lower than 9 on the Apgar test, having had language problems or a sibling with dyslexia). This relationship should be taken into account in the study of learning difficulties as a potential indicator to predict later reading development and even the presence of developmental dyslexia.

## 1. Introduction

Developmental dyslexia is defined as a neurocognitive learning disorder affecting reading and writing characterised by the persistent and specific non-fluid identification of words in children who do not present sensorial deficits, intellectual disabilities, other mental or neurological disorders, psychosocial adversity, or inadequate educational experiences [1]. It seems to originate from an alteration in neurodevelopment, and at the present time, its aetiology is recognised as being multifactorial, involving multiple interacting risk factors, which may be genetic or environmental [1,2]. These risk factors alter the development of multiple neural systems and the cognitive functions necessary for normal development, thereby producing the behavioural symptoms which define DD [3]. Given that additional environmental factors increase the risk of developing this reading disability and its related neuropsychological components, it can be supposed that different ecological niches moderate the strength of the genetic signal in accordance with a more articulated framework of gene-environment (GxE) interaction and interdependence [4,5]. Thus, the learning of reading is a complex issue as it requires mastery of multiple processes and the involvement of many factors.

According to Cuetos [6], the first task to master when acquiring this skill is to identify the letters of the alphabet and to learn their corresponding sounds. Therefore, due to the fact that children with reading disabilities take longer to learn the names of the letters, alphabet knowledge (AK) is considered to be one of the major predictors of developmental dyslexia (DD) in stages prior to the formal learning of reading [7,8,9]. However, although knowing the letters can be considered the strongest predictor of reading ability at the preschool level [10], it may also be ephemeral as many of the difficulties experienced in preschool are resolved at the beginning of primary education [10,11]. As a result, the precision of this prediction factor diminishes at the end of the preschool stage. However, the possibility should also be taken into account that some difficulties may not be detected in the early stages of education or that they emerge as the level of academic difficulty increases.

Phonological awareness (PA) is another reliable factor in predicting reading disabilities in different languages at the preschool stage [8,12,13,14,15], and it may have an incidental influence in the development of reading abilities [16]. The contribution of PA to the decoding of words has been well established [17]. Indeed, it is one of the reading predictors that has received the most attention in the study of the early phases of reading with some authors considering it to be the best predictor of literacy learning and DD [18,19]. Transparency in the spelling of each language can also have an influence, since it has been found that the more consistent the spelling, the lower the chances of predicting PA [20,21]. This is due to the fact that reading accuracy and PA can easily be acquired, even in dyslexic children. Thus, it may be of use in predicting individual variation in reading in the initial phases, although it ceases to be an adequate indicator in the early years of primary school due to the early ceiling effect [22,23].

At the same time, other authors maintain that the main component for the prediction of learning to read and the differential diagnosis of DD is the explicit and conscious manipulation of segments (phonemes or syllables) rather than tasks which only require the identification of a linguistic unit [24]. Thus, PA should not be considered to be a unique phenomenon, but rather as a continuum in which different levels of difficulty (syllable awareness, intrasyllabic awareness, lexical awareness and phonemic awareness) are involved. These difficulties may emerge in different orders during linguistic development, depending on the individual’s experience [25]. In general, the abilities of phonemic awareness and, in particular, phoneme deletion, are considered to be the most consistent predictor of learning how to read and DD [16,19,20,21,26,27].

In addition to AK and PA, there is evidence to support the fact that naming speed (NS) is another important indicator in predicting later acquisition of reading skills from an early age and in discriminating between typical and atypical (e.g., dyslexic) readers [20,21,23,28,29,30,31,32]. This is due to the fact that one of the characteristics of such children tends to be their slow reading speed (i.e., low performance in tasks concerning the naming of visual stimuli) [9,22]. Kim and Pallante [33] observed that individual differences in speed when naming letters and in phonetic segmentation were the only predictors in word reading that were maintained over time for Spanish children in the preschool stage. Furthermore, Gómez-Velázquez et al. [34] found that rapid naming of letters was the best predictor for reading performance, as it correctly identified 63% of children who subsequently presented difficulties in reading speed, which has been considered to be the definitive feature of DD in Spanish.

Therefore, letter knowledge can be considered to be the strongest predictor of reading ability in the preschool stage [10]. Furthermore, although PA and NS are both strong predictors of reading fluency and DD [21,35] in the primary education stage in transparent languages, NS barely plays a role in the initial phases, in which PA seems to be more significant, although it does play a more important role in later stages of education [12,22,35].

Different studies on the origins and development of DD reveal the need to consider, along with specific psycholinguistic predictors, different biopsychosocial risk factors (maternal, perigestational, neonatal, health and family-related) due to their influence on the development of reading skills and on dyslexia [36,37,38,39,40,41,42]. In this regard, certain determining environmental factors of a biopsychosocial nature are considered to increase the risk of developing reading difficulties [40], and it is estimated that 30% of individual differences in reading can be attributed to environmental factors [10]. However, although the contribution of the environment to the origins and development of DD has not been sufficiently analysed, and the literature on this topic is scarce and fragmented [43], there is evidence to support the fact that the perinatal and postnatal periods can be critical in terms of reading ability [38,43,44]. Different perigestational and neonatal complications, such as iodine deficiency during pregnancy, prematurity, insufficient intrauterine growth, hyperbilirubinaemia and neonatal hyperglycaemia, among others, have been described and may significantly affect neurodevelopment, causing atypical cognitive development that could lead to the presence of reading difficulties [38,45,46,47,48,49].

Several studies [39] argue that a delay in motor and language skills are risk factors for DD in infancy. Thus, the Diagnostic and Statistical Manual of Mental Disorders (DSM-5) [1] considers that a Specific Learning Disorder is frequently preceded in the preschool years by delays in attention, language, or motor skills that can persist and co-exist with the same disorder. More specifically, it seems that the presence of language difficulties at the beginning of the formal reading learning process is a particular risk factor for poor language and literacy outcomes [8,50]. Thompson et al. [8] studied children from at-risk families aged between 3.5 and 8 years, with the aim of examining which factors would predict the individual risk of DD. The results indicated that motor skills only contributed to prediction models for DD at 6 years of age and were not very relevant in earlier stages of development. Hence, some authors consider motor skills to be relatively unrelated to reading development and to have a low value in terms of the prediction of DD [51].

Last of all, a child’s early environment, to a large degree, is conditioned by cultural and family characteristics, which have a significant impact on his/her brain development during their childhood and adolescence [52]. In this regard, many studies have indicated that there is a link between both decoding and reading comprehension and aspects of the child’s family environment [37,40,53]. However, at present, there is no agreement on which environmental risk factors are clearly involved in the aetiology of this disorder [44]. Thus, examining the role which can be played by the environment in the neurobiological circuits of reading is of great importance in order to provide a much-needed perspective of how variables other than genetics influence the reading skills of children [43]. Therefore, the aim of this study is to identify the principal psycholinguistic abilities involved in the early reading performance and to analyse their relationship with biopsychosocial risk factors.

## 2. Materials and Methods

### 2.1. Participants

A total of 110 participants aged between 4 and 7 years of age, enrolled in state-run schools and from different socio-economic environments, participated in this study. The mean age was 5.9 years old (SD = 1.04), with a range of 4.0–7.91 years. 48.6% were male (mean age = 5.92 years old (SD = 1.10)) and 51.4% were female (mean age = 5.89 years old (SD = 0.98)) (see Table 1).

### 2.2. Instruments

The following instruments were used to obtain the data for the study:

- *Batería de Inicio a la Lectura* (BIL 3–6) [54]. This is a test battery which evaluates, via 15 tests and 143 items, linguistic skills related to the initial learning of reading in children aged between 3 and 6 years old.
- The Dyslexia Screening Test-Junior (DST-J) [55]. A Spanish adaptation of this test, aimed at students between the ages of 6 to 11 years old, was employed. It contains 13 tests that evaluate different aspects to enable the detection of dyslexia and plans for school programmes to assist in child development.
- NEPSY-II (the updated and modified version of the NEPSY instrument) [56]. This tool consists of 32 tests and 4 delayed memory tasks designed to evaluate neuropsychological development in children between the ages of 3 and 16.11 in six cognitive domains (attention and executive function, language, memory and learning, sensorimotor skills, social perception and visuospatial processing).
- Early Reading Performance Screening Scale. This is an ad hoc scale for use with children aged between 4 and 7 years old, which includes, in accordance with the literature, the main psycholinguistic abilities involved in the early reading performance. It consists of three tasks: (i) *Alphabet knowledge*: children’s knowledge of the names of the letters of the alphabet and/or their sounds, being the maximum number of correct answers 27. (ii) *Phonological awareness*: the ability to recognise and use the sounds of spoken language, including: syllable awareness (evaluating the capacity to identify the number of syllables which make up a word); intrasyllabic awareness (the ability to identify rhyming words); and phonemic awareness (the ability to identify the initial, intermediate or final phonemes of a word, via tasks involving identification, addition or omission). The number of items varies in each of the three subtests, with 53 being the maximum number of correct answers (22 in syllable awareness, 5 in intrasyllabic awareness and 26 in phonemic awareness). (iii) *Naming speed*: evaluating the ability to name a series of visual stimuli (drawings, colours, letters and numbers) as quickly as possible; the time (seconds) taken to complete the test is recorded.
- A Questionnaire on Biopsychosocial Risk. This tool is an ad hoc questionnaire designed to gather information on the participants’ mothers. It consists of different questions on the pregnancy, the child’s development and certain socio-family characteristics. It is structured in seven parts: Pregestational, Perigestational, Intrapartum, Neonatal, Medical, Developmental and Family.

### 2.3. Procedure

Different state-run schools were contacted in order to request their collaboration in the recruitment of participants. The schools’ support and guidance departments were asked to gain authorisation from the mothers of pupils aged between 4 and 7 years of age for their children to participate in the study. The following exclusion criteria were employed: intellectual, sensorial and motor disabilities or other mental or neurological disorders or psychosocial adversity. The conditions under which their data would be collected were specified (confidentiality, anonymity and agreement) and compliance with Organic Law 3/2018, of 5 December, on Protection of Personal Data and Guarantee of Digital Rights [57] was ensured.

The collection of the children’s data was carried out in the schools by a member of the research team who went to the schools and followed the procedure that was recommended by the head of the school. The instruments used were selected based on the children’s age: the *Batería de Inicio a la Lectura* (BIL 3–6) in preschool education and the Dyslexia Screening Test-Junior (DST-J) in primary education. The NEPSY-II and the Early Reading Performance Screening Scale were applied, both in the preschools and in the primary schools. The Questionnaire on Biopsychosocial Risk was sent to the mothers to be completed in their homes.

### 2.4. Analysis

The data analysis was carried out using IMB SPSS Statistics (v. 24, SPSS Inc., Chicago, IL, USA). Firstly, the basic psychometric properties from the Early Reading Performance Screening Scale were obtained. To achieve this, its internal consistency was measured using Cronbach’s alpha for the total Scale, with a value equal to or greater than 0.70 being considered acceptable [58]. The homogeneity index for the tests of the Scale, the alpha when eliminating each test and the correlation between the tests were also calculated. In addition, the Spearman correlation between the tests of the Scale and the NEPSY-II, DST-J and BIL-3 tests were calculated to obtain evidence of convergent validity.

The frequencies (percentage) for the biopsychosocial risk factors and the descriptive statistics for the psycholinguistic abilities involved in the early reading performance were determined. The relationship between both groups of variables and the correlation between the different dimensions of the Questionnaire on Biopsychosocial Risk were obtained via the Spearman correlation coefficient.

In both cases, the Holm method [59] was applied for adjusting *p*-values in order to reduce the potential inflation of type I errors due to multiple comparisons.

The association between the biopsychosocial risk factors which correlated significantly with psycholinguistic skills was studied via the Chi-squared test and the Phi correlation coefficient for dichotomous variables.

## 3. Results

The results of the internal consistency and convergent validity analyses of the Early Reading Performance Screening Scale confirmed its good psychometric properties. In addition, the results obtained with regard to the correlations between the tests of which it consists were significant and, in general, high or appropriate. As far as the psycholinguistic abilities described in the literature are concerned, these were evaluated via different tools, with consistent scores being obtained. In all of the tests applied, a greater degree of mastery could be observed as the participants’ age increased. Furthermore, a significant relationship was found between different psycholinguistic skills identified as predictors of early reading performance and certain biopsychosocial risk factors.

### 3.1. The Psychometric Properties of the Early Reading Performance Screening Scale

The Cronbach´s alpha value for the total Scale was 0.88 and the homogeneity indices can be considered adequate, oscillating within a range of 0.647 to 0.870, with the exception of the case of intrasyllabic awareness, the value of which was 0.510. The descriptive statistics, the homogeneity for each test and the alpha if a test is deleted can be seen in Table 2.

The highest correlations among the tests of the Scale were obtained between PA and Speed in naming letters and among these and AK (see Table 3)

The convergent validity was established by correlating the tests of the Scale with different tests from the NEPSY-II, DST-J (primary education sample) and the BIL 3–6 (preschool sample) aimed at evaluating similar or theoretically related constructs with the aptitudes evaluated by the Scale (see Table 4). Focusing on the highest values, the correlation between the alphabet knowledge tests of the scale and of the BIL 3–6 (0.930) is worthy of mention.

The Intrasyllabic Awareness test correlated significantly with two rhyming tasks, from the DST-J (0.601) and the BIL 3–6 (0.646). The strongest correlation found between Syllable Awareness and the NEPSY-II was with Speeded Naming (0.767) and Phonological Processing (0.753). Phonemic Awareness correlated highly with different tests from NEPSY-II, particularly Memory for Names Delayed (0.886), Phonological Processing (0.851) and Speeded Naming (0.808), and with the Rhyme (0.704) and Phonemic Segmentation (0.688) tests from the DST-J.

A consistent pattern was also found in the relationship between Phonological Awareness and different tests from the other instruments, especially NEPSY-II and DST-J. As far as NEPSY-II is concerned, the correlations obtained with Memory for Names Delayed (0.880), Phonological Processing (0.858) and Speeded Naming (0.832) stand out. In the case of the DST-J, it is worth noting the relationship with Rhyme (0.747), One Minute Writing (0.653) and Phonemic Segmentation (0.619) and, in the BIL 3–6, the correlation with Alphabet Knowledge (0.654) and Isolating Syllables and Phonemes (0.571).

The results were similar for Naming Speed. The strongest correlations were obtained with the tests from the NEPSY-II, particularly Memory for Names Delayed (−0.776), Word Generation (foods) (−0.738) and Speeded Naming (−0.733). Thus, it is worth mentioning the association found with Two Minute Spelling (−0.711) of the DST-J, and with Isolating Syllables and Phonemes (−0.625) of the BIL 3–6.

### 3.2. Performance in Psycholinguistic Abilities and the Presence of Biopsychosocial Risk

The results obtained following the application of the Scale have made it possible to observe an increase in the number of correct answers as age progresses (see Table 5). Thus, in the Early Reading Performance Screening Scale, while at 4 years of age the mean number of correct answers in Alphabet Knowledge was 9.35, participants of the age of 7, had a mean score of 20.87. As far as Phonological Awareness is concerned, the most obvious progress made was in the tests on Phonemic Awareness, in which the older age group obtained more than twice as many correct answers as those who were younger. Naming Speed increased progressively, with the older age group requiring half the time than that employed by the younger age group to perform the test (19.42 vs. 47.68 sg.) These results are consistent with those obtained in the tests for Phonological Processing and Speeded Naming from NEPSY-II. In the first case, the scores were very similar for the groups of 4 and 5-year-olds (16.33 and 18.88, respectively), with a noticeable improvement in performance beginning from the age of 6. Conversely, with regard to Naming Speed, there is a noticeable increase of correct answers between 4 and 5 years of age, with progression slowing down after that age.

The aptitudes related with reading and writing assessed by the DST-J improved in all cases between 6 and 7 years of age. The most evident progress was noted in the tests of One Minute Reading, Two Minute Spelling and One Minute Writing, in which the group of 7-year-olds practically doubled or even tripled the scores of the 6-year-old participants. Nonsense Passage Reading also underwent a notable improvement (40.71 vs. 56.66).

The children aged from 4 to 5 years old obtained scores that were much more homogenous in the different BIL 3–6 tests. The scores were close to the maximum in Articulation (12.76 and 14.50 out of 15, respectively) and Counting Syllables (11.06 and 11.27 out of 14, respectively). Meanwhile, in the rest of the tests there was considerable room for improvement.

The presence of biopsychosocial risk factors in the sample were, in general terms, reduced. Nevertheless, in the Pregestational dimension, the following aspects are worthy of note: advanced age of the mother (40% older than 34 years of age); a waiting time for pregnancy greater than 12 months (13.7%); problems in getting pregnant (10.1%); and all aspects related with the need for any type of fertility treatment (8.3%). In addition, 8.3% were at risk of miscarriage in this pregnancy, and 16.5% suffered a miscarriage at any point in time. The risk factors related to the Perigestational dimension were weight gain not in accordance with that which is recommended (55.8%), the presence of diseases during pregnancy (29.1%), substance abuse (particularly, tobacco in 19%) and the intake of medication (17.8%).

The Intrapartum dimension was characterised by the need for the purposeful breaking of the amniotic sac (27.5%), caesarean section (23.5%) and the use of medical instruments during the birth (18.3%). All of this can be related to the fact that 17.4% of the births took longer than 12 h once the contractions had begun, even though in very few cases the infant was breech (3.9%), or the birth consisted of multiple infants (1%).

The data for the Neonatal dimension confirmed a high percentage of premature births (44.0%) and 16.8% of new-borns with a birth weight considered to be outside of the adequate range, even though only 4.9% presented any type of growth restrictions. Neonatal illnesses had no (or very little) incidence, with only 9.5% of the children requiring urgent care and 10.5% suffering hyperbilirubinemia, according to the data provided by the mothers based on medical reports. Moreover, the score for the Apgar test (1 and 5 min) was less than 9 in 18.6% and in 5.1% of the cases, respectively. Finally, breastfeeding, considered to be a developmental protection factor, was the option chosen by 83.8% of the mothers, either exclusively or in combination with artificial milk.

The Medical dimension gathers information on sensory problems, conditions and surgical interventions. Hearing and vision were normal, with the exception of a few cases of impaired vision (12.4%). 18.1% suffered from asthma/allergies and 6.8% from recurring ear infections. The majority (85.0%) did not require any kind of surgery. The most common risk factor in the Developmental dimension was difficulties in language development (31.4%), with problems with motor skills being much less frequent (10.5%). Only 12% of the children had been diagnosed with some type of disorder, although 22.1% of the mothers had expressed some concern regarding their child’s development (mainly relating to attention deficit, impulsiveness and emotional problems). Last of all, in the Family-related dimension, the existence of family members with specific learning disorders (20.8%) and siblings with ADHD (7.8%) can be highlighted. In 2% of the cases, a sibling had dyslexia or a language disorder.

### 3.3. Relationship between Psycholinguistic Skills Predictive of Early Reading Performance and Biopsychosocial Risk Factors

Significant correlations were found between different psycholinguistic abilities and certain biopsychosocial risk factors (having had hyperbilirubinemia, having obtained a score lower than 9 on the Apgar test, having had language problems or a sibling with dyslexia) (see Table 6).

Problems in language development from the Developmental dimension was the factor which presented a significant relationship with the greatest number of abilities, related with Articulation (−0.648) in the BIL 3–6 and with Body Part Naming and Identification (−0.589) and Verbal Fluency (−0.446) in NEPSY-II.

Secondly, the relationship between the Apgar score and the tests of Repetition of Nonsense Words (−0.632) in NEPSY-II and Syllable Omission (−0.610) in BIL 3–6 should be highlighted. Last of all, the relationships that should be mentioned are those between the BIL 3–6 Vocabulary test and having had hyperbilirubinemia (−0.423) and the Backwards Digit Span test of the DST-J and having a sibling with dyslexia (−0.351).

The results obtained in the analysis carried out between the four biopsychosocial risk factors which correlated significantly with the psycholinguistic abilities included in the reading tests did not show a significant association with each other.

The results of the analysis of correlations carried out between the dimensions of the questionnaire also demonstrated a lack of association with each other, and although a significant relationship was found between the Medical and Neonatal and Developmental dimensions, the values obtained were lower than 0.3 in all cases.

## 4. Discussion

The purpose of this study was to analyse the relationship between early predictive abilities of developmental dyslexia and biopsychosocial risk factors.

There is a progressive improvement in the tests that evaluate AK, PA and NS as the age of the participants increases. The scored obtained in Alphabet Knowledge and in Intrasyllabic Awareness at the age of 7 were very high. Therefore, it can be considered that both skills are mastered at that age. Syllables are already identified with considerable accuracy at 5 years of age with few changes from that age on, and the learning of Phonemic Awareness improves, particularly from 6 years of age. These results coincide with those obtained in other studies which have confirmed the developmental character of phonological knowledge. Thus, at 4 years of age, syllables are the units that children can recognise and manage most easily (Syllabic Awareness). At the age of 5, they can think about intrasyllabic items, and around 6–7 years of age (at the beginning of the formal learning reading stage), they begin to think about phonemes (Phonemic Awareness) [26,60].

The most prevalent biopsychosocial risk factors in the sample were related to the mothers: inappropriate weight gain during pregnancy (55%); pregnancy after 34 years of age (40%); and consumption of medication during gestation (29.1%). As far as children are concerned, the following factors can be highlighted: prematurity (44%), the purposeful breaking of the amniotic sac (27.5%) and the need for a caesarean (23.5%). Other noteworthy items include the mothers’ preoccupations (22.1%) regarding their children’s evolutionary development and the presence of a family member with a Specific Learning Disorder (20.8%).

Regarding the relationship between predictive abilities of DD and the biopsychosocial risk factors, the results reveal that psycholinguistic skills are mainly related to developmental factors, although neonatal and family factors also have a role to play.

At the developmental level, language delays are a risk factor in obtaining a worse score on the Early Reading Performance Screening Scale, revealing that development during infancy is also a risk factor for the development of learning to read, as has been demonstrated in other research [9,11,36,39,61]. Indeed, DD is frequently preceded, in the preschool years, by language delays which can persist and coexist with this disorder over time [1,39,61,62].

There is much evidence that speech and language are closely associated with literacy [63] and that children with a genetic risk of DD tend to present deficiencies in these areas in the preschool years [61]. Therefore, these early speech perception abilities predict later literacy in such children [61,62]. Thus, it is proposed that deficiencies in this area can act as barriers to progress in other skills, such as phonological development and literacy [61].

In the first place, it should be noted that children with a sibling with dyslexia have worse psycholinguistic abilities. This fact is supported by different studies in which a familial history of DD has been identified as one of the strongest risk factors in literacy outcomes [7,8,11,31,42,43,61]. In this regard, it is estimated that up to 70–75% of the phenotypic variation in DD can be explained by genetic factors [64], in which the presence of a family history of DD would considerably increase the risk of a child experiencing reading difficulties [10,61]. However, the large number of studies which have noted how reading skills are closely related to the cultural and educational atmosphere in the child’s family environment should also be taken into account [37,40,41,42,53].

In relation to the biopsychosocial factors associated with the Neonatal dimension, our data reveals that the presence of jaundice at birth significantly correlates to various early predictors of DD. These results are in accordance with other studies that show the relative contribution of hyperbilirubinemia to the presence of later onset neurodevelopment disorders [47,65], although this association seems to be complex and presents a certain degree of controversy [45]. The fact that the level of bilirubin which can cause brain damage is still unknown should be taken into account, although a common point of reference is TSB< 26mg/dl [66], in accordance with Bhutani and Johnson-Hamerman [45]. However, there is a general consensus that there is no direct or precise relationship between moderate or serious hyperbilirubinemia and the overall neurological outcome as, in many cases, the affected children have multiple risk factors for the deterioration of neurological development, including prematurity, perinatal complications and haemolytic illness.

The low scores in the Apgar can be described along the same lines. Our results are both preliminary and surprising. It is important to recognise the limitations of the Apgar score, which is an expression of the physiological state of the baby at a given moment and includes subjective components. It is well known that there are numerous factors which can influence this score, such as the sedation or anaesthetic administered to the mother, congenital malformations, gestational age, traumas and variability among observers [67]. Therefore, further studies are required to clarify this relationship.

## 5. Conclusions

Different psycholinguistic abilities, such as alphabet knowledge and naming speed of letters and numbers, are clearly associated with reading disabilities and disorders, with a higher correlation being verified between them. Furthermore, different biopsychosocial factors have also demonstrated a significant relationship with psycholinguistic abilities. The results reveal a correlation between psycholinguistic abilities of a neonatal and family-related nature and, particularly, with developmental factors. However, it should be stressed that these are preliminary results which should be contemplated taking into consideration the size and the characteristics of the sample, since no major literacy difficulties were detected in the participants, and the presence of biopsychosocial risk factors were, in general terms, reduced. In future studies, an increased sample size and the incorporation of participants with a diagnosis of reading disabilities or dyslexia will, without doubt, enable the confirmation of a relationship between early predictors of DD and biopsychosocial risk factors that may facilitate the early identification of this disorder.

## Figures and Tables

**Table 1:** Characteristics of the sample.

	Female				Male			
	Mean	SD	n	%	Mean	SD	n	%
Age	5.89	0.98			5.92	1.10		
4 years			8	14.3			9	17.0
5 years			6	10.7			7	13.2
6 years			26	46.4			16	30.2
7 years			16	28.6			21	39.6
Total			56	100.0			53	100.0

**Table 2:** Mean, standard deviation (SD), homogeneity and alpha if test is deleted.

Test	Correct Answers		Errors		Time (s)		Homogeneity	Alpha If Test Is Deleted
	Mean	SD	Mean	SD	Mean	SD		
AK	17.32	5.66	4.93	6.18			0.849	0.853
IA	3.94	1.32	1.07	1.34			0.510	0.888
SA	13.55	3.04	3.81	2.51			0.647	0.880
PA	16.88	6.04	8.22	6.26			0.824	0.854
SND					28.36	8.44	0.831	0.844
SNC					31.97	12.30	0.782	0.843
SNL					29.68	20.60	0.868	0.878
SNN					22.95	11.95	0.870	0.833

Note: AK = Alphabet knowledge, IA = Intrasyllabic awareness, SA = Syllable awareness, PA = Phonemic awareness, SND = Speed in naming drawings, SNC = Speed in naming colours, SNL = Speed in naming letters, SNN = Speed in naming numbers.

**Table 3:** Correlations between the tests of the Early Reading Performance Screening Scale.

Test	AK	IA	SA	PA	SND	SNC	SNL	SNN
AK	-							
IA	0.469 *	-						
SA	0.592 *	0.403 *	-					
PA	0.718 *	0.518 *	0.682 *	-				
SND	−0.581 *	−0.394 *	−0.545 *	−0.653 *	-			
SNC	−0.638 *	−0.419 *	−0.576 *	−0.649 *	0.679 *	-		
SNL	−0.762 *	−0.536 *	−0.676 *	−0.818 *	0.750 *	0.709 *	-	
SNN	−0.675 *	−0.396	−0.623 *	−0.700 *	0.727 *	0.632 *	0.814 *	-

Note: AK = Alphabet knowledge, IA = Intrasyllabic awareness, SA = Syllable awareness, PA = Phonemic awareness, SND = Speed in naming drawings, SNC = Speed in naming colours, SNL = Speed in naming letters, SNN = Speed in naming numbers. Naming speed is measured in seconds: more time in seconds is related to fewer correct answers on the other reading tests. * *p* < 0.05.

**Table 4:** Correlations between the tests of the Early Reading Performance Screening Scale, NEPSY-II, the DST-J and BIL 3–6.

Test	Test of the Early Reading Performance Screening Scale					
	AK	IA	SA	PA	PhonA	NS ^c^
**NEPSY-II**						
Comprehension of Instructions		0.450		0.547 *	0.558 *	−0.644 *
Word Generation (animals)			0.607 *	0.660 *	0.683 *	−0.656 *
Word Generation (foods)			0.710 *	0.684 *	0.739 *	−0.738 *
Phonological Processing	0.668 *	0.404	0.753 *	0.851 *	0.858 *	
Repetition of Nonsense Words			0.414	0.562	0.532	−0.471
Oromotor Sequences	0.730 *	0.700 *		0.654 *	0.746 *	−0.731 *
Speeded Naming ^a^			0.767 *	0.808 *	0.832 *	−0.733 *
Memory for Names (delayed)			0.544	0.886 *	0.880 *	−0.776 *
Sentence Repetition	0.686 *	0.584 *		0.736 *	0.723 *	
**DST-J**						
Rapid Naming ^b^				−0.461	−0.432	0.484
One Minute Reading	0.493		0.262	0.646 *	0.588 *	−0.529
Phonemic Segmentation				0.688 *	0.619 *	−0.468
Rhyme		0.601 *	0.418	0.704 *	0.747 *	−0.731 *
Two Minute Spelling	0.281			0.551	0.452 *	−0.711 *
Backwards Digit Span				0.576	0.450	
Nonsense Passage Reading	0.498	0.298	0.251	0.597 *	0.606 *	−0.556
One Minute Writing				0.606 *	0.653	−0.631 *
Verbal Fluency					0.305	−0.444
**BIL 3–6**						
Articulation				0.598 *	0.426	−0.546
Sequential Auditory Memory	0.550		0.298	0.480	0.419	
Perception			0.397		0.308	−0.506
Alphabet Knowledge	0.930 *	0.420	0.466	0.624 *	0.654 *	
Word Recognition				0.516	0.427	−0.371
Word Counting	0.497	0.430	0.308	0.292	0.369	−0.508
Isolating Syllables and Phonemes	0.696 *			0.560	0.571 *	−0.625 *
Reading Functions					0.217	
Grammatical Structures			0.546		0.407	−0.543
Rhyme	0.506	0.646 *	0.345		0.472	−0.470
Counting Syllables				0.522	0.508	
Omission of Syllables			0.574 *	0.369	0.481	
Vocabulary					0.165	−0.250

Note: AK = Alphabet knowledge, IA = Intrasyllabic awareness, SA = Syllable awareness, PA = Phonemic awareness, PhonA = Phonological awareness, NS = Naming speed. ^a^ Correct answers are recorded, ^b^ Time and errors are recorded, ^c^ Time is recorded. Regarding naming speed, more time on the Early Reading Performance Screening Scale and more time and errors on the DST-J are related to fewer correct answers on the other test. * *p* < 0.05.

**Table 5:** Mean and standard deviation (SD) in the Tests of the Early Reading Performance Screening Scale, the DST-J, the BIL 3–6 and the NEPSY-II in accordance with participants’ age.

Tests	Maximum Score Possible	4 Years	5 Years	6 Years	7 Years
		(n = 17)	(n = 13)	(n = 42)	(n = 37)
		M (SD)	M (SD)	M (SD)	M (SD)
Scale of Early Reading Performance predictors					
Alphabet knowledge	27	9.35 (4.72)	16.3 (6.98)	18.34 (4.11)	20.87 (1.23)
Intrasyllabic Awareness	5	2.70 (1.40)	4.32 (1.01)	3.90 (1.26)	4.43 (1.09)
Syllable Awareness	18	9.64 (1.32)	11.38 (2.75)	13.83 (2.72)	15.78 (1.41)
Phonemic Awareness	26	8.94 (3.45)	14.53 (4.87)	16.80 (5.48)	21.56 (2.70)
Naming Speed ^c^	-	47.68 (9.45)	32.39 (8.21)	26.93 (8.07)	19.42 (3.10)
**NEPSY-II**					
Comprehension of Instructions	33	19.82 (3.34)	23.00 (1.95)	24.62 (2.50)	24.60 (3.98)
Word Generation (animals)	-	8.40 (2.96)	13.57 (2.15)	15.13 (2.75)	20.00 (6.60)
Word Generation (foods)	-	7.20 (2.62)	8.86 (0.90)	15.25 (3.85)	16.40 (6.50)
Phonological Processing	22 (3–4 years) 45 (5–16 years)	16.33 (1.72)	18.88 (4.39)	28.17 (5.95)	34.80 (3.19)
Repetition of Nonsense Words	46	22.67 (14.01)	33.78 (9.32)	35.88 (5.46)	41.00 (4.85)
Oromotor Sequences	70	30.00 (0.00)	40.40 (12.49)	54.75 (6.30)	57.00 (5.43)
Speeded Naming ^a^	600	27.27 (15.47)	80.86 (2.32)	133.0 (2.77)	134.0 (1.41)
Sentence Repetition	34	22.50 (3.44)	24.83 (3.01)	29.50 (2.20)	29.75 (4.57)
**DST-J**					
Rapid Naming ^b^	-			45.88 (9.99)	44.63 (9.74)
One Minute Reading	-			26.71 (15.87)	51.08 (22.40)
Phonemic Segmentation	12			8.21 (2.75)	9.33 (2.35)
Rhyme	8			4.64 (1.01)	6.17 (1.47)
Two Minute Spelling	32			8.14 (2.88)	17.00 (2.86)
Backwards Digit Span	14			3.07 (1.14)	3.58 (0.99)
Nonsense Passage Reading	58			40.71 (19.48)	56.66 (2.83)
One Minute Writing	-			2.57 (0.98)	9.00 (3.79)
Verbal Fluency	25			6.71 (1.86)	10.00 (2.92)
**BIL**					
Articulation	15	12.76 (2.31)	14.50 (1.17)		
Sequential Auditory Memory	35	13.41 (2.27)	13.67 (2.05)		
Perception	22	11.75 (4.97)	14.75 (6.51)		
Alphabet Knowledge	24	12.82 (5.83)	18.42 (7.28)		
Word Recognition	10	8.18 (1.67)	9.00 (1.12)		
Counting words	6	2.59 (0.87)	3.33 (1.44)		
Isolating Syllables and Phonemes	8	5.41 (1.12)	6.25 (1.87)		
Reading Functions	5	3.24 (1.03)	4.00 (0.74)		
Grammatical Structures	6	3.71 (1.45)	5.42 (0.79)		
Rhyme	12	6.71 (2.69)	9.55 (1.86)		
Counting Syllables	14	11.06 (1.35)	11.27 (2.94)		
Omission of Syllables	5	2.82 (1.29)	3.58 (1.38)		

Note: ^a^ Correct answers are recorded, ^b^ Time and errors are recorded, ^c^ Time is recorded.

**Table 6:** Correlations between biopsychosocial risk factors and psycholinguistic abilities.

	Psycholinguistic Abilities	Biopsychosocial Risk Factors			
		1	2	3	4
**Early Reading Performance Screening Scale**	Alphabet knowledge	−0.198 *			−0.218 *
	Intrasyllabic Awareness	−0.225 *			−0.197 *
	Phonemical Awareness				
	Naming Speed	−0.219 *			
**NEPSY-II**	Comprehension of Instructions				−0.259 **
	Repetition of Nonsense Words		−0.632 *		
	Body Part Naming and Identification			−0.589 *	
	Word Generation (foods)			−0.446 *	
	Memory for Designs (Delayed)				
**DST-J**	Phonemic Segmentation				
	Nonsense Passage Reading				
	Backwards Digit Span				−0.351 *
**BIL 3–6**	Grammatical Structures	−0.423 *			
	Vocabulary	−0.331 *			
	Omission of Syllables		−0.610 *		
	Articulation			−0.648 **	

Note: 1 = Presented hyperbilirubinemia; 2 = Having obtained a score less than 9 on the Apgar test (minute 1); 3 = Having presented problems in language development; 4 = Has a sibling with dyslexia. * *p* < 0.05, ** *p* < 0.01.

## Data Availability

The data (anonymised, with no identifying information) are available upon reasonable request to the corresponding author.

## References

[1] American Psychiatric Association (APA) (2013). Diagnostic and Statistical Manual of Mental Disorders.

[2] Peterson R.L., Pennington B.F. (2015). Developmental dyslexia. Annu. Rev. Clin. Psychol..

[3] Pennington B.F. (2006). From single to multiple deficit models of developmental disorders. Cognition.

[4] Battaglia M. (2012). Challenges in the appraisal and application of gene? Environment interdependence. Eur. J. Dev. Psychol..

[5] Rutter M. (2007). Gene-environment interdependence. Dev. Sci..

[6] Cuetos F. (2009). Dislexias evolutivas: Un puzzle por resolver. Rev. Logop. Foniatr. Audiol..

[7] Poulakanaho A., Ahonen T., Aro M., Eklund K., Leppänen P.H., Poikkeus A.M., Tolvanen A., Torppa M., Lyytinen H. (2007). Very early phonological and language skills: Estimating individual risk of reading disability. J. Child Psychol. Psychiatry.

[8] Thompson P.A., Hulme C., Nash H.M., Gooch D., Hayiou-Thomas E., Snowling M.J. (2015). Developmental dyslexia: Predicting individual risk. J. Child Psychol. Psychiatry.

[9] Torppa M., Lyytinen P., Erskine J., Eklund K., Lyytinen H. (2010). Language development, literacy skills, and predictive connections to reading in Finnish children with and without familial risk for dyslexia. J. Learn. Disabil..

[10] Ozernov-Palchik O., Yu X., Wang Y., Gaab N. (2016). Lessons to be learned: How a comprehensive neurobiological framework of atypical reading development can inform educational practice. Curr. Opin. Behav. Sci..

[11] Snowling M.J., Melby-Lervåg M. (2016). Oral language deficits in familial dyslexia: A meta-analysis and review. Psychol. Bull..

[12] Furnes B., Samuelsson S. (2011). Phonological awareness and rapid automatized naming predicting early development in reading and spelling: Results from a cross-linguistic longitudinal study. Learn. Individ. Differ..

[13] Macdonald H.H., Sullivan A.L., Watkins M.W. (2013). Multivariate screening model for later word reading achievement: Predictive utility of prereading skills and cognitive ability. J. Appl. Sch. Psychol..

[14] Ozernov-Palchik O., Norton E.S., Sideridis G., Beach S.D., Wolf M., Gabrieli J.D., Gaab N. (2017). Longitudinal stability of pre-reading skill profiles of kindergarten children: Implications for early screening and theories of reading. Dev. Sci..

[15] Papadimitriou A.M., Vlachos F.M. (2014). Which specific skills developing during preschool years predict the reading performance in the first and second grade of primary school?. Early Child Dev. Care.

[16] Melby-Lervåg M., Lyster S.A.H., Hulme C. (2012). Phonological skills and their role in learning to read: A meta-analytic review. Psychol. Bull..

[17] Lipka O. (2017). Reading fluency from grade 2–6: A longitudinal examination. Read. Writ..

[18] Cuetos F., Suárez-Coalla P., Molina M.I., Llenderrozas M.C. (2015). Test para la detección temprana de las dificultades en el aprendizaje de la lectura y escritura. Pediatr. Aten. Prim..

[19] González Seijas R.M., López Larrosa S., Vilar Fernández J., Rodríguez López-Vázquez A. (2013). Estudio de los predictores de la lectura. Rev. Investig. Educ..

[20] Caravolas M., Lervåg A., Defior S., Seidlová Málková G., Hulme C. (2013). Different patterns, but equivalent predictors, of growth in reading in consistent and inconsistent orthographies. Psychol. Sci..

[21] Landerl K., Ramus F., Moll K., Lyytinen H., Leppänen P.H., Lohvansuu K., O’Donovan M., William J., Bartling J., Bruen J. (2013). Predictors of developmental dyslexia in European orthographies with varying complexity. J. Child Psychol. Psychiatry.

[22] Defior S., Serrano F. (2011). Procesos fonológicos explícitos e implícitos. Lectura y Dislexia. Rev. Neuropsicol. Neuropsiquiatr. Neurocienc..

[23] Suárez-Coalla P., García-de-Castro M., Cuetos F. (2013). Variables predictoras de la lectura y la escritura en castellano. Infanc. Aprendiz..

[24] Bordoy Castro S. (2016). De la Teoría Fonológica a la Identificación Temprana y el Diagnóstico Diferencial de la Dislexia Evolutiva. Ph.D. Thesis.

[25] Aguilar M., Marchena E., Navarro J.I., Menacho I. (2011). Niveles de dificultad de la conciencia fonológica y aprendizaje lector. Rev. Logop. Foniatr. Audiol..

[26] Defior S., Serrano F. (2011). La conciencia fonémica aliada de la adquisición del lenguaje escrito. Rev. Logop. Foniatr. Audiol..

[27] Shanahan T., Lonigan C.J. (2010). The National Early Literacy Panel: A summary of the process and the report. Educ. Res..

[28] González R.M., Cuetos F., Vilar J., Uceira E. (2015). Efectos de la intervención en conciencia fonológica y velocidad de denominación sobre el aprendizaje de la escritura. Aula Abierta.

[29] Kirby J.R., Ball A., Geier B.K., Parrila R., Wade-Woolley L. (2011). The development of reading interest and its relation to reading ability. J. Res. Read..

[30] López-Escribano C., Sánchez-Hípola P., Suro Sánchez J., Leal Carretero F. (2014). Comparative Analysis of Rapid Automatized Naming Studies in Spanish and Reading Acquisition and Reading Difficulties. Univ. Psychol..

[31] Lundetræ K., Thomson J.M. (2018). Rhythm production at school entry as a predictor of poor reading and spelling at the end of first grade. Read. Writ..

[32] Rabazo M.J., García M., Sánchez S. (2016). Exploración de la conciencia fonológica y la velocidad de nombrado en alumnos de 3º Educación Infantil y 1º de Educación Primaria y su relación con el aprendizaje de la lectoescritura. Rev. INFAD Psicol..

[33] Kim Y.S., Pallante D. (2012). Predictors of reading skills for kindergartners and first grade students in Spanish: A longitudinal study. Read. Writ..

[34] Gómez-Velázquez F.R., González-Garrido A.A., Zarabozo D., Amano M. (2010). La velocidad de denominación de letras: El mejor predictor temprano del desarrollo lector en español. Rev. Mex. Inv. Educ..

[35] Vaessen A., Blomert L. (2010). Long-term cognitive dynamics of fluent reading development. J. Exp. Child Psychol..

[36] Dilnot J., Hamilton L., Maughan B., Snowling M.J. (2017). Child and environmental risk factors predicting readiness for learning in children at high risk of dyslexia. Dev. Psychopathol..

[37] Hamilton L. (2013). The Role of the Home Literacy Environment in the Early Literacy Development of Children at Family-Risk of Dyslexia. Ph.D. Thesis.

[38] Hay I., Hynes K.L., Burgess J. (2019). Mild-to-moderate gestational iodine deficiency processing disorder. Nutrients.

[39] Helland T., Jones L.Ø., Helland W. (2017). Detecting Preschool Language Impairment and Risk of Developmental Dyslexia. J. Res. Child. Educ..

[40] Mascheretti S., Bureau A., Battaglia M., Simone D., Quadrelli E., Croteau J., Cellino M.R., Giorda R., Beri S., Maziade M. (2013). An assessment of gene-by-environment interactions in developmental dyslexia-related phenotypes. Genes Brain Behav..

[41] Sun Z., Zou L., Zhang J., Mo S., Shao S., Zhong R., Juntao K., Lu X., Miao X., Song R. (2013). Prevalence and associated risk factors of dyslexic children in a middle-sized city of China: A cross-sectional study. PLoS ONE.

[42] Van Bergen E., de Jong P.F., Plakas A., Maassen B., van der Leij A. (2012). Child and parental literacy levels within families with a history of dyslexia. J. Child Psychol. Psychiatry.

[43] Becker N., Vasconcelos M., Oliveira V., Dos Santos F.C., Bizarro L., De Almeida R.M., De Salles J.F., Carvalho M.R.S. (2017). Genetic and environmental risk factors for developmental dyslexia in children: Systematic review of the last decade. Dev. Neuropsychol..

[44] Mascheretti S., Andreola C., Scaini S., Sulpizio S. (2018). Beyond genes: A systematic review of environmental risk factors in specific reading disorder. Res. Dev. Disabil..

[45] Bhutani V.K., Johnson-Hamerman L. (2015). The clinical syndrome of bilirubin-induced neurologic dysfunction. Semin. Fetal Neonatal Med..

[46] Horowitz-Kraus T. (2018). The early signs of reading difficulties at school can start with foetal growth restriction. Acta Paediatr..

[47] Hokkanen L., Launes J., Michelsson K. (2014). Adult neurobehavioral outcome of hyperbilirubinemia in full term neonates—A 30 year prospective follow-up study. PeerJ..

[48] Kaiser J.R., Bai S., Gibson N., Holland G., Lin T.M., Swearingen C.J., Mehl J., ElHassan N.O. (2015). Association between transient newborn hypoglycemia and fourth-grade achievement test proficiency: A population-based study. JAMA Pediatr..

[49] Kovachy V.N., Adams J.N., Tamaresis J.S., Feldman H.M. (2015). Reading abilities in school-aged preterm children: A review and meta-analysis. Dev. Med. Child Neurol..

[50] Duff F.J., Reen G., Plunkett K., Nation K. (2015). Do infant vocabulary skills predict school-age language and literacy outcomes?. J. Child Psychol. Psychiatry.

[51] Sellés P., Martínez T., Vidal-Abarca Gámez E. (2012). Controversia entre madurez lectora y enseñanza precoz de la lectura: Revisión histórica y propuestas actuales. Aula Abierta.

[52] Fox S.E., Levitt P., Nelson C.A. (2010). How the timing and quality of early experiences influence the development of brain architecture. Child Dev..

[53] Taylor H.G., Klein N., Anselmo M.G., Minich N., Espy K.A., Hack M. (2011). Learning problems in kindergarten students with extremely preterm birth. Arch. Pediatr. Adolesc. Med..

[54] Sellés P., Martínez T., Vidal-Abarca E., Gilabert R. (2019). Batería de Inicio a la Lectura.

[55] Fawcet A.J., Nicolson R.I. (2016). Test para la Detección de la Dislexia en Niños.

[56] Korkman M., Kirk U., Kemp S. (2014). NEPSY-II: Manual Clínico y de Interpretación.

[57] Organic Law 3/2018, of 5 December, on Protection of Personal Data and Guarantee of Digital Rights. Boletín Oficial del Estado, 294, of 6 December of 2018, 119778 a 119857. (2018). https://www.boe.es/eli/es/lo/2018/12/05/3.

[58] Hays R.D., Anderson R., Revicki D. (1993). Psychometric considerations in evaluating health-related quality of life measures. Qual. Life Res..

[59] Holm S. (1979). A simple sequentially rejective multiple test procedure. Scan J. Stat..

[60] Sellés P., Giménez T. (2014). Secuencia evolutiva del conocimiento fonológico en niños prelectores. Rev. Logop. Foniatr. Audiol..

[61] Carroll J.M., Mundy I.R., Cunningham A.J. (2014). The roles of family history of dyslexia, language, speech production and phonological processing in predicting literacy progress. Dev. Sci..

[62] Boets B., Vandermosten M., Poelmans H., Luts H., Wouters J., Ghesquière P. (2011). Preschool impairments in auditory processing and speech perception uniquely predict future reading problems. Res. Dev. Disabil..

[63] Snowling M.R., Hulme C. (2011). Evidence-based interventions for reading and language difficulties: Creating a virtuous circle. Br. J. Educ. Psychol..

[64] Poelmans G., Buitelaar J.K., Pauls D.L., Franke B. (2011). A theoretical molecular network for dyslexia: Integrating available genetic findings. Mol. Psychiatry.

[65] Maimburg R.D., Bech B.H., Væth M., Møller-Madsen B., Olsen J. (2010). Neonatal jaundice, autism, and other disorders of psychological development. Pediatrics.

[66] Jain M., Bang A., Tiwari A., Jain S. (2017). Prediction of significant hyperbilirubinemia in term neonates by early non-invasive bilirubin measurement. World J. Pediatr..

[67] American Academy of Pediatrics, Committee on Fetus and Newborn, The American College of Obstetricians and Gynecologists, Committee on Obstetric Practice (2015). The Apgar Score. Obstet. Gynecol..

